# Spontaneous Epidural Hemorrhage in Sickle Cell Disease, Are They All the Same? A Case Report and Comprehensive Review of the Literature

**DOI:** 10.1155/2019/8974580

**Published:** 2019-06-26

**Authors:** Biplab Saha, Aditi Saha

**Affiliations:** ^1^Division of Pulmonary and Critical Care Medicine, Albany Medical Center, Albany, NY, USA; ^2^Department of Medicine, Saint Barnabas Medical Center, Livingston, NJ, USA

## Abstract

Trauma to the skull causing injury to the middle meningeal artery, middle meningeal vein, or dural venous sinuses is responsible for most cases of epidural hemorrhage (EDH). Spontaneous EDH is a rare entity in clinical practice. Common causes include sinusitis, coagulation abnormalities, dural metastasis, and Langerhans cell histiocytosis. Isolated nontraumatic EDH is an exceedingly rare complication of sickle cell disease (SCD). We report a case of spontaneous EDH in a patient with SCD and review the world literature regarding this rare entity. A 20-year-old African American female with sickle cell disease presented with vaso-occlusive crisis. About 24 hours after hospital admission, the patient had sudden deterioration of her mental status. An emergent CT scan of the head revealed a large right-sided frontoparietal epidural hematoma with midline shift, subfalcine, and uncal herniation. The patient underwent emergent hematoma evacuation but died 24 hours after surgery.

## 1. Introduction

Sickle cell disease (SCD) is caused by a single amino acid substitution on the beta globin chain resulting in the propensity of the hemoglobin molecule to polymerize in deoxygenated state. The abnormal polymerization is responsible for subsequent RBC injury, hemolysis, microvascular injury, and classical acute and chronic manifestations of the disease. Neurologic manifestations secondary to SCD are common and affect about 35% of patients [1]. Although reports vary [2], existing data suggest that 54% of these patients suffer from ischemic strokes and 34% from intracranial bleeding [1]. Intracerebral, subarachnoid, intraventricular, subdural, and epidural bleeds have been reported, with subarachnoid hemorrhage being the most common, especially in young adults [1–3]. Isolated nontraumatic spontaneous epidural hematoma (EDH) is an exceedingly rare complication of SCD. Other reported etiologies of spontaneous EDH are infectious [4–6], coagulation abnormalities associated with end stage renal disease and hemodialysis [7–9], dural metastasis [10–13], and Langerhans cell histiocytosis [14–16]. We present a case of spontaneous EDH in a sickle cell patient suffering from vaso-occlusive crisis and review the world literature regarding the rare entity.

## 2. Case Presentation

A 20-year-old African American female with a history of sickle cell disease (HbSS) and multiple previous admissions for vaso-occlusive crisis (VOC) presented to the hospital with severe generalized pain throughout her body. The patient was in severe distress. Her blood pressure was 155/101 mmHg, pulse 117 beats per minute, temperature 37.6 C, respiratory rate 25 breaths per minute, and oxygen saturation 98% on room air. Physical examination revealed poor bilateral air entry on lung auscultation due to splinting and an ejection systolic murmur over the aortic area. Mild-to-moderate tenderness was present over the extremities on palpation. Neurological examination was normal. Blood work showed leukocytosis, 12,800 with 53% neutrophil, 32% lymphocyte, and 1% band, hemoglobin 7.5 gm/dL, hematocrit 22.3%, platelet 181,000/dL, reticulocyte count 13%, lactate dehydrogenase 1144 IU/L, normal blood urea nitrogen, creatinine, and serum electrolyte studies. Liver function tests were normal except a total bilirubin level of 16.2 mg/dL. Chest X-ray was normal. The patient was started on IV hydration; analgesia was achieved by IV narcotics.

**Figure 1:**
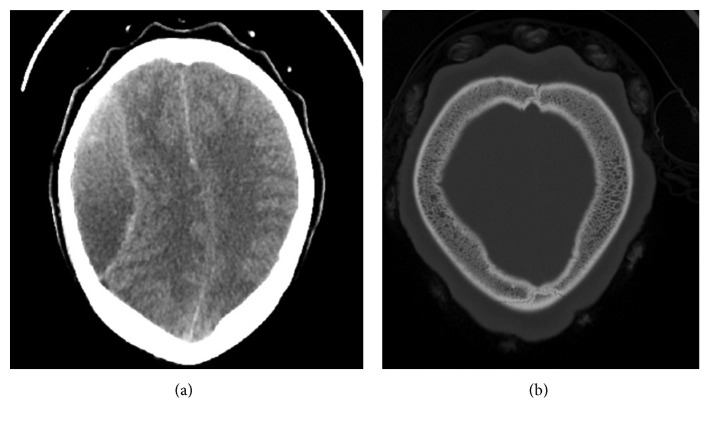
(a) Mixed density right frontoparietal epidural hematoma with midline shift. (b) Bony window showing massive expansion of the diploic bone from extramedullary hematopoiesis.

About 24 hours after hospital admission, the patient suddenly became unresponsive. Naloxone failed to improve her mental status. An emergent CT scan of the head revealed a large right-sided frontoparietal epidural hematoma with midline shift, subfalcine, and uncal herniation. No subgaleal or subperiosteal collection was noted. There was no noticeable bone infarction overlying the hematoma (Figure 1).

**Figure 2:**
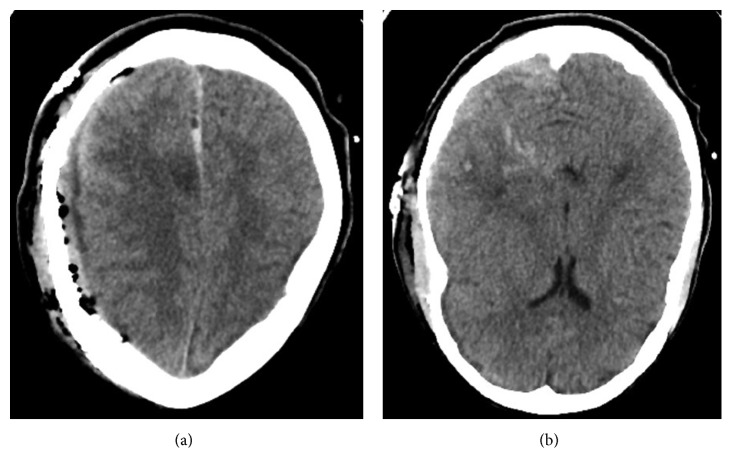
(a) Postoperative changes following hematoma evacuation with a small collection in the subdural space and significant improvement of midline shift (b) Punctate hemorrhage in the right temporal lobe postop.

Laboratory data at this time demonstrated a platelet of 45,000/dL, prothrombin time 19.7 Seconds, INR 1.7, activated partial thromboplastin time 43 seconds, and a fibrinogen level of 96 mg/dL, consistent with a diagnosis of disseminated intravascular coagulation (DIC). The patient was emergently taken to the operating room for hematoma evacuation (Figure 2). Intraoperatively, no obvious bony abnormality was noted. Exchange transfusion was performed. The patient, however deteriorated, became hypotensive requiring multiple vasopressors and eventually died about 24 hour after surgical intervention from DIC.

Total 31 cases. R, right; L, left; N/S, not specified; B/L, bilateral; VOC, vaso-occlusive disease; FP, frontoparietal; PT, parietotemporal; DIC, disseminated intravascular coagulation; POA, present on admission; BS, bone scan; IO, intraoperatively.

## 3. Discussion

**Figure 3:**
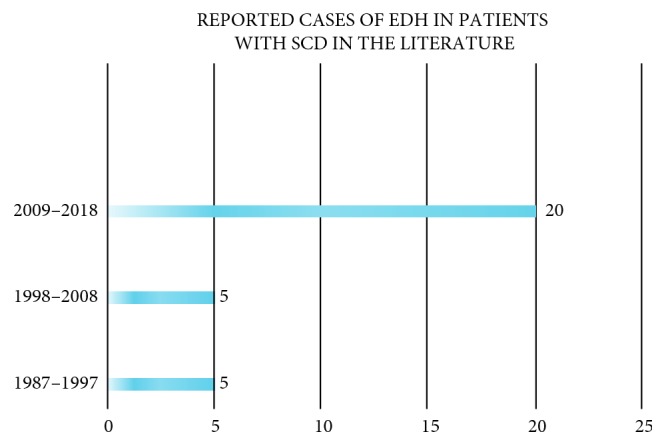
Reported cases of EDH in patients with SCD in the literature.

We have described a patient with SCD suffering from spontaneous EDH is the setting of VOC. Including the present case, we have identified 31 cases described in the literature, which are shown individually in Table 1. Since first reported about 3 decades ago, the reporting incidence has increased precipitously in the last decade possibly due to increased awareness, radiologic advancement, and more opportunities for publication rather than actual increase in incidence (Figure 3).

Most reported cases comprised males 25/31 (80%). The age ranged from 2–35 years (median 16.5) and 7–19 years (median 16), respectively, for males and females. 26/31 (84%) had SCD (17 patients with reported homozygosity and 9 known cases without specified haplotype). Other cases included 3 patients with HbSC disease, 1 with HbSD, and sickle thalassemia each.

Headache was present on admission in 37% (11/30), whereas symptoms consistent with VOC, primarily pain crisis, were the initial complaint in 40% (12/30). Other presenting symptoms were eye swelling, proptosis, seizure, and coma. In patients who presented with a headache, EDH was present on admission in 82% of the time. Patients who presented with VOC, progressed to have EDH within 6–120 hours, with a median of 24 hours after hospital admission.

Unilateral EDH was more common 20/31 (65%). Subgaleal hemorrhage was concurrently present in 9/30 (30%) of patients and associated with bone infarction in (7/9) 78% of cases. 48% of patients (15/31) with EDH showed evidence of overlying bony infarction. The overall mortality among the reported cases was 23% (7/30). Interestingly, patients with evidence of overlying bone infarction had a survival of 100% and patients with DIC had a mortality of 100%.

The most common cause of EDH is trauma causing injury to the middle meningeal artery, middle meningeal vein, the diploic vein, or the dural venous sinuses [44]. Spontaneous nontraumatic EDH is a rare manifestation of SCD and variant sickle cell syndromes. The pathophysiology of this rare occurrence is not completely understood.

Most reported cases in the literature are males. Whether any gender specific etiology is responsible for this discrepancy is not known. Low steady-state hemoglobin and high leukocyte count are known risk factors for hemorrhagic stroke in patients with SCD [1]. Whether this is also true for EDH is not clear. In the landmark study by Ohene-Frempong et al., hemorrhagic strokes were more prevalent in the adult SCD patients in the age range of 20–30 years [1]. However, EDH appears to be more prevalent in the adolescent age group.

Three pathophysiologic explanations have been proposed over the years for the causation of this rare entity: (1) Vaso-occlusion of the haematopoietically active calvarial diploic bone resulting in bone infarction and subsequent leaking of blood and proteinaceous material in the subperiosteal, epidural, or subgaleal space [38–41, 43, 45]. (2) Acute rapid expansion of hematopoiesis with resultant microfracture of already thinned inner cortex and extravasation of blood and hematopoietic tissue [24, 28, 36]. (3) Sludging of sickle cells in the diploic veins hampering venous drainage and oozing of blood due to vascular injury and elevated back pressure. A combination of different mechanisms could also be responsible. Presence of coagulopathy or platelet dysfunction worsens hematoma expansion and portends a dire clinical outcome.

Patients admitted with VOC developed EDH, did so with a median of 24 hours, suggesting early deterioration is expected in this patient population. Patients who suffered from concurrent bony infarction or subgaleal hemorrhage had a survival rate of 100%. This may indicate that vaso-occlusive etiology of the EDH carries better prognosis. This would also explain presence of EDH with contralateral subgaleal hemorrhage as skull bone infarction could be diffused [40]. The identification of bone infarction can be challenging. MRI appears to be the most sensitive tool. CT scan has low yield especially in the acute phase of the disease [18]. Infarcted bone can appear normal intraoperatively although thinning of the inner cortex is frequently seen. In 3 out of 7 patients who succumbed to their disease, an MRI ruled out any bony infarction. In the other 4 cases, no bone damage was noted by CT scan or on intraoperative inspection. We propose that patients without any obvious bony disruption and infarction, most likely suffer from microfracture of the inner table and extrusion of blood and hematopoietic cells in the epidural space due to rapid expansion of hematopoiesis. Presence of coagulopathy as in DIC makes the bleeding significantly worse. Patients who suffered from spontaneous EDH and suffered from DIC had a 100% mortality.

## 4. Conclusion

The understanding regarding this clinical entity is rapidly evolving in the era of advanced technology and improved awareness among healthcare providers. We believe this review will shed more light on the clinical as well as the pathophysiological aspect of the disease process and help in rapid identification of patients at risk of deterioration and formulate management plan accordingly.

## Figures and Tables

**Table 1:** 

Case report	Year	Age (years)	Sex	Haplotype	Presentation	Identification of EDH after hospitalization	Location of EDH	Subgaleal hemorrhage	Subperiosteal collection	Skull infarction	DIC present	Outcome
Current case	2019	19	Female	HbSS	VOC	24 hours	R FP	No	No	No (CT/IO)	Yes	Died
Komarla et al. [17]	2018	18	Female	Unspecified	VOC	24 hours	R parietal	No	No	Yes (MRI)	N/S	Survived
		17	Male	Unspecified	Headache	POA	Bifrontal	Yes	Yes	Yes (MRI)	N/S	Survived
Banerjee et al. [18]	2018	Teenage	Male	HbSS	VOC	6 hours	L frontal	No	No	No (MRI)	Yes	Died
Moyen et al. [19]	2018	13	Male	HbSS	Seizure	Imaging not performed till day 8	R FP	No	N/S	No (MRI)	No	Died
Mishra et al. [20]	2017	18	Male	HbSS	VOC	120 hours	R parietal	Yes	No	Yes (IO)	No	Survived
Gajjar and Gupta [21]	2015	20	Male	Unspecified	Headache	POA	L PT	No	No	No (CT)	No	Survived
Hettige et al. [22]	2015	7	Female	HbSS	Coma	POA	B/L parietal	No	No	No (CT/IO)	Yes	Died
Yogarajah at al. [23]	2015	19	Male	HbSC	VOC	24 hours	R PT	No	No	No (CT/IO)	No	Survived
N'dri Oka et al. [24]	2015	19	Male	HbSC	Headache	POA	Occipital	Yes	No	No (CT)	No	Survived
Ilhan et al. [25]	2014	15	Male	HbSS	Headache	POA	Right frontal	Yes	No	Yes (MRI)	No	Survived
Serarslan et al. [26]	2014	19	Female	HbSS	Headache	POA	L FP	No	No	No (CT/IO)	No	Survived
Page et al. [27]	2014	20	Male	HbSS	VOC	48 hours	L frontal	Yes	No	Yes (MRI)	No	Survived
		7	Female	HbSS	Coma	POA	R temporal	No		Yes (MRI)	No	Survived
Babatola et al. [28]	2012	18	Male	HbSS	Headache	POA	R Frontal	No	No	No (CT/IO)	No	Survived
Bolke and Scherer [29]	2012	19	Male	Unspecified	VOC	72 hours	L frontal	No	No	No (CT/IO)	No	Died
Patra et al. [30]	2012	13	Male	Unspecified	Headache	POA	B/L parietal	No	No	No (CT)	No	Survived
Arends et al. [31]	2011	19	Male	HbSC	Headache	POA	R parietal	No	No	Yes (MRI)	No	Survived
Sangle et al. [32]	2011	15	Male	Unspecified	Headache	12 hours	Bifrontal	No	No	No (MRI)	No	Died
Azhar [33]	2010	12	Male	HbSD	VOC	24 hours	L frontal	No	No	N/S (CT)	N/S	Survived
Dahdaleh et al. [34]	2009	18	Male	Unspecified	VOC	12 hours	B/L FP	Yes	No	No (CT/IO)	No	Survived
Kotb et al. [35]	2006	10	Male	Unspecified	Headache	N/S	Bifrontal	Yes	No	Yes (MRI)	N/S	Survived
Kalala Okito et al. [36]	2004	2	Male	HbSS	Coma	POA	R FT	No	No	N/S (IO)	N/S	Died
		12	Male	HbSS	VOC	N/S	L parietal	No	No	Yes (X-ray)	No	Survived
Ganesh et al. [37]	2001	11	Male	HbSS	Proptosis	POA	Bifrontal	Yes	No	Yes (BS)	N/S	Survived
Naran and Fontana [38]	2001	16	Male	Unspecified	Headache	? POA	R Frontal	No	No	Yes (MRI/BS)	No	Survived
Cabon et al. [39]	1997	14	Female	HbSS	Unknown	Unknown	Bifrontal	Unknown	Unknown	Yes	Unknown	Unknown
Resar et al. [40]	1996	14	Male	HbSS	VOC	48 hours	Left parietal and B/L frontal	Yes	Yes	Yes (MRI/BS)	No	Survived
Tony et al. [41]	1995	35	Male	Sickle- thalassemia	Proptosis	POA	Left frontal	No	No	Yes (BS)	No	Survived
Karacostas et al. [42]	1991	19	Male	HbSS	VOC	48 hours	Left frontal	No	No	No (CT)	No	Survived
Mallouh et al. [43]	1987	13	Male	HbSS	Eye swelling	POA	Bifrontal	No	No	Yes	N/S	Survived
